# National Sample Vital Registration System: A sustainable platform for COVID-19 and other infectious diseases surveillance in low and middle-income countries

**DOI:** 10.7189/jogh.10.020368

**Published:** 2020-12

**Authors:** Agbessi Amouzou, Almamy Kante, Ivalda Macicame, Adriano Antonio, Eduardo Gudo, Pedro Duce, Robert E Black

**Affiliations:** 1 Institute for International Programs, Department of International Health, Johns Hopkins Bloomberg School of Public Health, Baltimore, Maryland, USA; 2 Instituto Nacional de Saude, Maputo, Mozambique; 3 Instituto Nacional de Estatistica, Maputo, Mozambique

The COVID-19 pandemic raises the critical need for effective national surveillance systems, capable of detecting the onset of outbreaks rapidly but also sustainable platforms for mortality and cause of death (CoD) surveillance that allow rapid data collection to address questions during and after epidemics or crises. These pre-, during, and post-outbreak functions are necessary for effective responses. They are particularly needed in resource-constrained countries where health systems are limited. Low- and middle-income countries (LMIC) struggle to establish exhaustive surveillance platforms at community level for national response in real time. Systems such as Integrated Disease Surveillance and Response are mostly limited to health facilities. Beside, they do not generate standard mortality and CoD indicators over time. Many organizations, including the World Health Organization, react to the COVID-19 by developing tools to support countries with rapid mortality surveillance strategies. However, a significant challenge is the crucial lack of comparable historical data allowing an assessment of excess mortality due to the COVID-19 [1,2]. Well-designed sample vital registration systems (SVRS) offer rapid, and sustainable platforms for achieving the need for real-time data and the ability to nest data collection to respond to rising questions [3].

**Figure:**
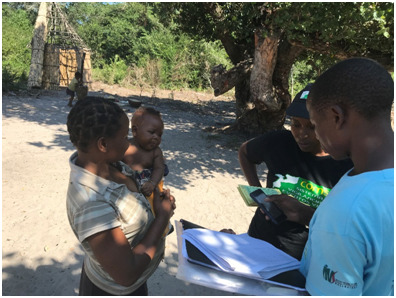
Photo: From COMSA Project showing interview of a mother with a baby. Data are collected directly on a phone (from the authors’ own collection, used with permission).

An SVRS uses a national random sample of communities to continuously track population and demographic events to measure multiple indicators including mortality and CoD. It constitutes a second alternative to exhaustive monitoring of the entire country population, as in civil registration and vital statistics (CRVS) systems. An SVRS fulfills the features needed in a multi-surveillance system. First, with most deaths occurring at the community level, it is an ideal platform for generating recent nationally representative mortality and cause-specific mortality rates. It allows an understanding of the seasonal patterns of deaths over time, thus permitting the measurement of any excess mortality due to temporary crises, as well as changes in CoD patterns by socio-demographic characteristics. Second, the system is often structured and staffed from communities to national levels, with long-term staff, making it ready for addition of data collection modules. Third, the system is ready for drawing subsample for specific purposes, including surveillance of diseases. The clusters will be well-established platforms for determining important epidemic transmission patterns, needed for adequate response. The rapid growth in digital solution offers a real-time data collection system solutions, allowing rapid data access and analysis. SVRS complements and reinforces other existing systems such as CRVS, Routine Health Information Systems, and household survey programs, and can serve to assess the accuracy, completeness and quality of these systems.

An SVRS can be developed and operationalized within a short period. However, to date, few LMICs are implementing it, as most continue to rely exclusively on household surveys. India initiated an SVRS in the 1970s and now covers 7597 clusters with over 7 million population. It produces reliable national and state representative mortality and CoD statistics [4]. The Bangladesh SRVS system was initiated in the 1980s in 103 communities and progressively increased to 2012 communities, covering about 700 000 people [5]. China’s SVRS integrated since 2013 two separate parallel systems and collects data from 601 clusters and about 324 million people [6]. In Africa, Mozambique launched an SVRS in 2017 with 700 clusters, about 900 000 people, producing mortality and CoD indicators [7] The government of Mozambique is implementing the SVRS with technical assistance from the Johns Hopkins University [8]. Using innovative digital tools, the system is set up for real-time data production, analysis, and release. The government is mobilizing the system to support its response to the COVID-19 pandemic, starting from the epicenter province in northern Mozambique.

SVRS systems have not received needed attention because the international community has hitherto focused on promoting household surveys. The emergence of outbreaks, such as the Ebola epidemic and the COVID-19 pandemic, which call for more permanent monitoring systems, provides further impetus for investment in sustainable and multi-purpose national SRVS.
